# A Comprehensive Analysis of the Relationship between Play Performance and Psychosocial Problems in School-Aged Children

**DOI:** 10.3390/children9081110

**Published:** 2022-07-24

**Authors:** Raúl Vigil-Dopico, Laura Delgado-Lobete, Rebeca Montes-Montes, José Antonio Prieto-Saborit

**Affiliations:** 1 Faculty Padre Ossó, University of Oviedo, 33008 Oviedo, Spain; raulvigil00@gmail.com (R.V.-D.); josea@facultadpadreosso.es (J.A.P.-S.); 2 Talionis Research Group, Centre for Information and Communications Technology Research (CITIC), Universidade da Coruña, 15008 A Coruña, Spain; rebeca.montes.montes@gmail.com

**Keywords:** psychosocial difficulties, daily performance, play performance, activities of daily living, occupational therapy, participation, school-aged, typically developing

## Abstract

During childhood, play contributes to the physical, emotional, cognitive and social development of infants and children and may enhance future mental health. The aim of this study was to examine the relationship between play performance factors and psychosocial problems in school-aged children. A total of 142 typical Spanish children aged 5 to 9 years were included. Play performance was measured with the My Child’s Play questionnaire, while the Strengths and Difficulties Questionnaire was used to evaluate internalizing and externalizing problems. The findings showed that personal, environmental and activity factors of play performance were associated with psychosocial problems and prosocial behavior in children. Moreover, children with high psychosocial difficulties reported significantly poorer play performance. As executive functioning during play was the factor that was most strongly associated with internalizing and externalizing psychosocial difficulties, it is possible that executive functions have a decisive role on both social cognition and self-regulation during play performance.

## 1. Introduction

Play is described as an intrinsically motivated, internally controlled and freely chosen activity that may involve exploration, fantasy, humor or risk taking [1,2,3]. Play performance encompasses a broad range of aspects that should be evaluated, including executive functioning, environment and material opportunities and uses, preferences and choices, and parent and peer relationships [4]. Therefore, it is necessary to consider the personal, environmental and activity-related aspects of play. Due to its multidimensional nature, play perception and conceptualization highly depend on sociocultural factors, which makes it a complex construct to assess [5].

The importance of play has been internationally highlighted and recognized, and it is a fundamental right of all children that should be ensured by the public health system [6]. As a main occupation during childhood, play greatly contributes to the physical, emotional, cognitive and social development of infants and children. Play activities allows children to develop the motor, processing, cognitive and social skills needed to participate in their daily present and future contexts [7,8,9,10]. Play activities provide a safe and controlled scenario for children to explore, express and learn to regulate their emotional and behavioral responses through interaction with caregivers and peers [11]. Moreover, promoting play and playfulness has proven to be a useful intervention strategy to help children to cope with severe mental health outcomes of trauma or acute medical procedures [12,13], and to enhance social and adaptative skills in children with intellectual, neurodevelopmental and learning disorders [14,15].

Play development and performance during middle childhood is of special relevance as children gain more independence and autonomy and social aspects and personal preferences become more important. Children between 5 and 9 years engage in more structured and organized play activities that usually involve higher psychosocial demands such as following rules or cooperating with peers. In addition, in middle childhood children become interested in achievement through play and form close friendships as they appreciate the social recognition that comes with successful performance of shared play projects [1,4,16]. Social skills and peer rejection are aspects of concern in middle childhood. Therefore, one of the most investigated benefits of play on childhood development is the influence of peer play ability on childhood mental health and psychosocial skills. Based on accumulative experimental and exploratory research [17,18,19,20], Zhao and Gibson [21] proposed a theoretical explanation of the relationship between peer play competence and mental health outcomes during early and middle childhood through social cognition and self-regulatory skills. According to this model, early peer play competence promotes socio-cognitive and self-regulation skills that will facilitate engagement in high-quality peer relational networks, which in turn contributes to a lower risk for later psychosocial issues such as anxiety and depression.

In fact, mental health in children and adolescents has become a public health issue of great importance. It is estimated that up to one in five children presents with mental health problems, and, more specifically, with internalizing and externalizing psychosocial problems such as anxiety, hyperactivity, depression or behavioural problems [22]. Although the relationship between social factors of play and psychosocial problems in children have been demonstrated, there are other play-related factors that may underly this link and that could contribute to further understanding it such as the environmental and executive functioning [23].

Overall, previous research has investigated the potential links between the social aspects of play and mental health outcomes in childhood and adolescence [21]. However, a comprehensively analysis of the relationship between the broad range of play performance aspects and psychosocial problems in typically developing children is lacking. It is yet unknown if the executive function, social and environmental aspects of play are equally associated with both externalizing and internalizing symptoms, as it could have important practical implications to developmental and health promotion programs. Moreover, children with psychosocial problems may display specific difficulties to engage in play activities, which would further limit their daily functioning. In addition, it is important to specifically examine this relationship in different cultural contexts, as children perform and participate in their daily occupations differently according to country and cultural setting [24].

Thus, the main aim of this study was to examine comprehensively the relationships between play performance factors and psychosocial problems in typically developing children.

## 2. Materials and Methods

### 2.1. Design, Ethical Considerations and Procedure

We conducted a cross-sectional study involving nine public elementary schools in Asturias (Spain). The study was approved by the Research Ethics Committee of the Principality of Asturias (code 2022.028) and was carried out in accordance to the Declaration of Helsinki. Parental consent was obtained from all the parents of the children enrolled in the study and from the schools’ boards.

To increase the representativity of the sample, rural and urban schools were invited to participate in the study. This region of Spain has a high percentage of people living in rural or semirural areas (4.0 and 14.9%, respectively) [25]; thus, two of the nine participating schools were gathered rural schools. As this kind of school is difficult to access, they were nonrandomly selected according to availability criteria. The remaining seven schools were located in urban settings and were randomly selected. All families of the students enrolled in the first four grades of elementary education of the participating schools received an online questionnaire between February and March 2022 that included the information form and informed consent, the Spanish versions of the My Child’s Play scale (MCP) and the Strengths and Difficulties Questionnaire (SDQ), and a socio-demographic questionnaire to gather information regarding child’s age, sex, clinical diagnosis of any neurodevelopmental or learning disorders, mother and father educational level, family structure, prematurity and presence of and age difference between siblings. Additionally, family socio-economic status (SES) was assessed using the family net income per month, which was calculated using the Spanish Public Income Indicator of Multiple Effects (IPREM) [26].

### 2.2. Sample Size and Participants

According to previous research, it was expected to find a medium-to-large correlation between play-related factors and psychosocial problems in children [19,20,21]. Thus, a minimum sample size of 138 children was used to measure a correlation of r ≥ 0.3 [27] with sufficient confidence and power (α = 0.05; power (1 − β) = 0.95). The child inclusion criteria were (a) aged between 5 and 9 years old; (b) no clinical diagnosis of intellectual, neurodevelopmental or learning disorder, and; (c) enrolled in a mainstream school in the 2021–2022 academic year. To ensure the eligibility criteria of the participants, the socio-demographic questionnaires were revised prior to data analysis, and those children who did not meet the criteria were excluded beforehand. A total of 168 parents returned the questionnaires, of which 12 children were excluded due to being younger than 5 years (*n* = 2) or older than 9 years (*n* = 10). In addition, 14 more children were discarded due to a parental report of diagnosed intellectual, neurodevelopmental or learning disorder. The final sample comprised 142 typically developing children: 58.5% girls; mean age (DT) 7.6 y (1.1 y; see Table 1). Participants had a mean of 0.9 siblings (SD = 0.6), and the reported age difference between participants and their siblings was 5.1 years (SD = 4.5).

### 2.3. Outcome Variables and Measures

#### 2.3.1. Play

Play performance was measured with the Spanish version of the MCP, a parent questionnaire designed to evaluate different aspects of play performance in children aged 4 to 9 [28]. The Spanish version of the MCP comprises 40 items that assess four factors: (1) play preferences and interpersonal relationships; (2) executive functions: flexibility and executive attention; (3) play characteristics, and; (4) environmental context). Each item is rated on a 5-point Likert scale, where higher scores reflect better play performance. In addition, a cut-off score of <142 on the MCP scale was indicative of atypical play associated with the presence of neurodevelopmental disorders (NDD-play) [29].

The MCP has acceptable internal consistency, construct validity and discriminant validity for Spanish children with and without neurodevelopmental conditions (internal consistency: Cronbach’s alpha = 0.695; construct validity: root mean square error of approximation = 0.023 (95% CI = 0.010–0.050), comparative fit index = 0.991, non-normed fit index = 0.990, root mean square of residuals = 0.048; discriminant validity: area under the curve = 0.876 (95% CI = 0.840–0.912)) [29].

#### 2.3.2. Psychosocial Difficulties

The Spanish version of the SDQ was used to measure the emotional, behavioral and social problems of the children [30,31,32]. It is a parent questionnaire that comprises 25 items divided into two externalizing factors: emotional symptoms and peer problems, and two externalizing factors: conduct problems and hyperactivity symptoms along with a fifth prosocial behavior factor. Each item is rated on a 3-point Likert scale, where higher scores are indicative of more psychosocial problems and less prosocial behavior. The scores of the first four factors are summed to obtain a total score of psychosocial problems. Children who score >90th percentile on the total problems scale are identified as having high psychosocial difficulties [33]. In this study, the Spanish percentiles developed by Barriuso-Lapresa et al. were used [34].

The SDQ has adequate internal consistency and construct validity in Spanish school-aged children (internal consistency: Cronbach’s alpha = 0.76; construct validity: root mean square error or approximation = 0.73 (95% CI = 0.068–0.077); comparative fit index = 0.93; goodness of fit index = 0.84) [32].

### 2.4. Data Analysis

Statistical analyses were conducted using IBM SPSS (version 25.0; IBM, Armonk, NY, USA) and JASP. Sample size was estimated using G*Power version 3.1.9.3. (https://doi.org/10.1016/j.paid.2016.06.069 (accessed on 10 January 2022). For descriptive purposes, frequency tables, mean, standard deviation, median and interquartile range were reported. Data were examined for normality using the Shapiro–Wilk test. As normality assumptions were not met for psychosocial measures, non-parametric bivariate analyses were used. Thus, Spearman correlations were conducted to examine the relationships between play performance domains, psychosocial problems and prosocial behavior. Differences in play performance among children with and without high psychosocial difficulties were analyzed using Student’s *t* tests, and the differences in psychosocial problems and prosocial behavior among children with and without NDD-play were analyzed using Mann–Whitney U tests. Cohen’s d and rank-biserial correlations were used to estimate effect sizes (small = 0.1, medium = 0.2 and large = 0.3) [27].

Finally, a linear regression model was performed to explore the predictive value of play performance factors over psychosocial problems. For all analyses, the alpha level was set to 0.05.

## 3. Results

### 3.1. Descriptive Results for Play and Psychosocial Problems

Descriptive findings for play performance and psychosocial problems are displayed in Table A1. Prevalence rates of NDD-play and high psychosocial difficulties according to the total scores on the MCP and SDQ scales were 10.6 and 7.8%, respectively.

### 3.2. Relationship between Psychosocial Problems and Play Performance

Small-to-large correlations were found between play performance factors and psychosocial problems (Table 2). All play factors were significantly associated with psychosocial problems and prosocial behavior except for “play environmental context” and for some psychosocial factors and “play preferences and interpersonal relationships”. Children with more psychosocial problems and poorer prosocial behavior displayed poorer play performance. Among play factors, executive functions during play showed the strongest correlations with externalizing symptoms and prosocial behavior (rho = 0.311–0.620).

Children with NDD-play showed higher psychosocial problems and less prosocial behavior than children with typical play, with a significant and large effect size (rank-biserial correlations = 0.309–0.688; see Table 3), whether children with high psychosocial difficulties reported poorer play performance, also with large effect sizes (d = 0.714–1.913; see Table 4) except for the environmental context factor (*p* > 0.05). Overall, children with NDD-play were 10 times more likely to display high psychosocial difficulties (OR = 10.1, 95% CI = 2.6–38.9; see Table 5).

Play performance factors explained almost half of the variance in psychosocial problems (adjusted R^2^ = 0.477, *p* < 0.001; see Table 6). Executive functioning during play accounted for most of the variance in psychosocial problems (B = −0.487 (95% CI = −0.626, −0.347)).

## 4. Discussion

The main objective of this study was to examine comprehensively the relationship between several personal, environmental and activity-related aspects of play performance and psychosocial problems in typically developing children. Our findings show that play performance and mental health display a complex relationship according to the factors involved.

Most play performance factors and general performance showed significant and medium-to-large associations with mental health and prosocial behavior. Specifically, executive functioning and play characteristics were associated with internalizing and externalizing problems, but play preferences and interpersonal relationships only showed a significant relationship with internalizing problems and prosocial behavior. Influence of peer social play on mental health during early and middle childhood has been previously explored. For instance, both cross-sectional and longitudinal data show that better peer and parent play ability at a young age is a protective factor against later internalizing and externalizing problems for the general population [11,21].

The potential link between deficits in non-play related executive functioning and internalizing and externalizing problems have been reported in previous research [35,36] but not within the specific context of play performance. The strong relationships found in this work regarding executive functioning during play and internalizing and externalizing psychosocial problems suggest that higher cognitive aspects of play may have a relevant role on the development of psychosocial problems, thus expanding and contributing to support the hypothesis of Zhao and Gibson [21] regarding the mediating role of social cognition and self-regulatory skills on the influence of peer play over mental health outcomes during middle childhood.

Interestingly, the environmental context of play performance was the play-related factor that displayed a weaker association with mental health in this sample, except for psychosocial general problems and peer problems. These findings partially agree with the work of Hinkley et al. [37] and Fyfe-Johnson et al. [23], who reported that context of play participation was a strong predictor of mental health and of cognitive, behavioral and social skills. However, the small correlations found in this study may be explained due to the fact that all children came from a specific region of Spain and all were enrolled in public schools with alike characteristics; thus, it is possible that environmental opportunities were similar for most of the sample.

Our results also showed that children who performed an atypical play activity indicative of NDD reported higher internalizing and externalizing problems and poorer prosocial behavior, while children with high psychosocial difficulties displayed significantly worse play performance factors except for the environmental context. It must be noted that the prevalence of risk of NDD found in this sample is similar to that reported in recent research regarding undiagnosed neurodevelopmental difficulties in Spanish school-aged children [38]. This is in line with previous studies that have found that children with NDD display more psychosocial problems than typically developing children [39,40,41], which further supports the use of tailored and individual intervention strategies that assess all the personal, environmental and activity-related aspects of daily functioning [42]. Children with NDD or other developmental difficulties often face more activity limitations and participation restrictions than their typically developing peers, not only in play activities but in other occupations as relevant as self-care, education and instrumental activities [43,44,45,46,47].

Given the relationship between early play performance and later mental health outcomes in childhood, it could be that early restrictions in play participation have an additional impact on daily functioning through internalizing and externalizing problems. However, it must be noted that, given the cross-sectional nature of this study, it may be possible that psychosocial problems in middle childhood are partially responsible for a reduced ability to perform play activities, especially regarding executive functioning skills. Both externalizing and internalizing symptoms have been previously reported to be associated with executive functioning and general cognitive in children [35,36]. Interestingly in this sample, play performance was not equally associated with externalizing and internalizing symptoms, as play preferences and interpersonal relationships were strongly correlated with internalizing problems but not with externalizing problems, while executive functioning during play displayed the strongest correlations with externalizing symptoms. This finding contributes to understanding how different aspects of play performance may enhance or hinder psychosocial well-being in children. For instance, children with externalizing problems could benefit from play activities with high executive functions demands, but they also could be more easily frustrated by these kinds of activities. Moreover, as longitudinal research suggests that lack of peer play skills is a prime stressor that eventually results in an increased risk of mental health problems during childhood [21], findings from this study indicate that it could also be that the presence of psychosocial problems contribute to restricting participation of play activities and to worsening the child’s performance in turn. In consequence, children showing externalizing symptoms, such as behavioral problems, may also display difficulties in executive functioning, while children with internalizing symptoms, such as anxiety or emotional and peer problems, could also display solitary play patterns, which may initiate a negative cycle that persists and worsens during late childhood and adolescence. Access to play should be guaranteed for all children, and thus it is necessary to detect those children at risk of restricted play participation to ensure that they are able to engage in those play activities that are beneficial for them.

Additionally, these findings suggest that typically developing school-age children face unmet needs regarding their play performance skills and psychosocial wellbeing. A high percentage of this sample was identified as having either atypical play associated with neurodevelopmental conditions or high psychosocial difficulties, which are two of the most prevalent problems during childhood and adolescence [38,48]. However, there is a systematic under-recognition of both mental and neurodevelopmental difficulties in children, which significantly limits prevention and treatment [38,49]. In this regard, schools may offer an excellent context to implement programs to enhance social and executive functioning development through playful interventions, as health professionals can identify and address performance and developmental difficulties early, thus preventing further, more pervasive, outcomes [50]. Accordingly, different school-based programs have been developed by psychologists and occupational therapists to promote different aspects of executive functioning and social skills, but also to improve mental health aspects through play activities, such as self-regulation [51,52,53,54,55,56].

Knowing which specific play-related factors correlate with psychosocial problems in children may help to tailor, play-based and evidence-driven interventions that comprehensively address the social and cognitive aspects of the proposed activities. However, occupational therapy-structured and evidence-based programs to promote mental health and development in schools are scarce in Spain although research indicates that they could be effective for promoting social play skills and self-regulation in typically developing school-aged children [57]. Using an interdisciplinary approach that includes both teachers and suitable health professionals may contribute to achieving adherence and positive outcomes, as teachers are able to identify social difficulties in children [58], and occupational therapists are experts regarding activity and performance analysis and play-based intervention [1,4,16]. In addition, poor parent–child play interactions and low playful environments have been found to be associated with psychosocial and behavioral problems in children [11,59]. Thus, enhancing family playfulness and involving parents using a family-centered approach may contribute to promote executive functioning and mental health in children, and to the generalization of those skills to other daily living contexts, such as home and community [58,60]. Mental health needs of children are usually unmet due to a lack of recognition or the unavailability of appropriate health care services [61,62]. As international recommendations advocate for a multitiered system of mental health support in schools [49,50], national policy makers can draw on research to implement context-specific and evidence-driven decisions. In the Spanish context, it is recommended that interdisciplinary, tailored, active and play-based methodologies to promote psychosocial and socio-emotional skills in mainstream schools be designed and carried out.

### Limitations and Future Research

The present study has some limitations that should be acknowledged. First, two of the participating schools were selected using a nonrandom criterion. Although this decision was made to ensure the participation of children from rural areas, future studies should include larger samples of randomly selected children living in rural and urban settings. Second, the cross-cultural nature of this work did not allow causal relationships between play performance and psychosocial problems to be established. Thus, longitudinal studies should be conducted. Given the findings regarding executive functioning during play, it would be advisable to expand this line of research during early and middle childhood.

## 5. Conclusions

The findings of this study corroborate and expand the current understanding of the relationship between play performance aspects such as play characteristics and social interactions and executive functioning, as well as both psychosocial difficulties and social behavior. To our knowledge, the present study is the first to comprehensively assess play performance and psychosocial problems in school-aged Spanish children, but it has implications for international research. As executive functioning during play was the individual factor that accounted for most of the variance in psychosocial difficulties, it is possible that executive functions have a decisive role in both social cognition and self-regulation during play performance. However, social aspects of play performance may be more strongly associated with emotional and peer problems. Overall, it can be concluded that play performance and psychosocial behavior in children display a complex and intertwined relationship, where different play factors are specifically associated with either externalizing or internalizing symptoms. Identification and intervention programs could take these findings into account to design precise, tailored strategies that specifically adjust to the children’s needs.

## Figures and Tables

**Table 1:** Sociodemographic data of the participants (*n* = 142).

Sociodemographic Characteristics	N (%)
Sex	
Boys	59 (41.5)
Girls	83 (58.5)
Questionnaire filled by	
Mother	131 (92.3)
Father	10 (7.0)
Other (legal guardian)	1 (0.7)
Siblings	
Only child	37 (26.1)
Has siblings	105 (73.9)
Family structure (including the child)	
Two members	5 (3.5)
Three members	35 (24.7)
Four members	86 (60.6)
Five members	16 (11.3)
Prematurity	
Preterm (<37 weeks of pregnancy)	12 (8.5)
Term (≥37 weeks of pregnancy)	130 (91.5)
Father educational level	
Incomplete first level studies	3 (2.1)
First level studies	22 (15.5)
Second level studies	85 (59.9)
University studies	32 (22.5)
Mother educational level	
Incomplete first level studies	1 (0.7)
First level studies	6 (4.2)
Second level studies	57 (40.1)
University studies	78 (54.9)
Family SES (IPREM) ^1^	
<2.5 IPREM	25 (17.6)
2.5–5.5 IPREM	78 (54.9)
5.5–7.5 IPREM	31 (21.8)
>7.5 IPREM	8 (5.6)

^1^ <2.5 IPREM ≤ 1412 euro per month; 2.5–5.5 IPREM = 1412–3107 euro per month; 5.5–7.5 IPREM = 3108–4237 euro per month; >7.5 IPREM ≥ 4237 euro per month.

**Table 2:** Correlations between play and psychosocial factors (*n* = 142).

Psychosocial Factors	Play General Performance	Executive Functions	Environmental Context	Play Characteristics	Play Preferences and Interpersonal Relationships
Psychosocial general problems	0.678 ***	0.620 ***	0.198 *	0.528 ***	0.331 ***
Emotional symptoms	0.473 ***	0.323 ***	0.071	0.322 ***	0.459 ***
Conduct problems	0.494 ***	0.530 ***	0.148	0.373 ***	0.134
Hyperactivity	0.456 ***	0.537 ***	0.092	0.445 ***	0.020
Peer problems	0.465 ***	0.311 ***	0.175 *	0.280 ***	0.426 ***
Prosocial behavior	0.404 ***	0.457 ***	0.080	0.285 ***	0.256 **

* *p* < 0.05; ** *p* < 0.01; *** *p* < 0.001.

**Table 3:** Psychosocial factors according to play performance (*n* = 142).

Psychosocial Factors	Typical Play M (SD)	NDD-Play M (SD)	*p* Value	Rank-Biserial Correlation
Psychosocial general problems	8.0 (5.1)	15.0 (4.8)	<0.001	0.688
Emotional symptoms	2.0 (2.1)	4.0 (2.5)	0.001	0.493
Conduct problems	1.3 (1.4)	3.6 (2.3)	<0.001	0.588
Hyperactivity	3.7 (2.5)	5.4 (3.4)	0.049	0.309
Peer problems	0.9 (1.2)	2.0 (1.8)	0.011	0.382
Prosocial behavior	8.7 (1.5)	6.9 (7.5)	0.008	0.408

**Table 4:** Play performance according to psychosocial difficulties (*n* = 142).

Play Factors	No Psychosocial Difficulties M (SD)	High Psychosocial Difficulties M (SD)	*p* Value	Cohen’s d
Play general performance	158.4 (11.5)	140.6 (7.3)	<0.001	1.580
Executive functions	43.5 (5.0)	33.9 (4.7)	<0.001	1.913
Environmental context	36.9 (4.0)	35.6 (3.1)	0.281	0.340
Play characteristics	42.1 (4.3)	38.4 (4.5)	0.007	0.862
Play preferences and interpersonal relationships	36.0 (4.2)	32.8 (6.7)	0.024	0.714

**Table 5:** Presence of psychosocial difficulties in children with typical and atypical play performance (*n* = 142).

	No Psychosocial Difficulties N (%)	High Psychosocial Difficulties N (%)	X^2^	*p* Value
Play			15.363	<0.001
Typical	121 (92.4)	6 (54.5)		
NDD	10 (7.6)	5 (45.5)		

**Table 6:** Linear regression for psychosocial problems (*n* = 142).

Predictors	B	95% Confidence Interval		*p* Value
		Lower Limit	Upper Limit	
Executive functions	−0.487	−0.626	−0.347	<0.001
Environmental context	−0.108	−0.277	0.061	0.208
Play characteristics	−0.282	−0.456	−0.108	0.002
Play preferences and interpersonal relationships	−0.133	−0.291	0.025	0.099

## Data Availability

The data are not publicly available due to containing information that could compromise the privacy of research participants.

## Appendix A

**Table A1:** Descriptive results for the MCP and the SDQ (*n* = 142).

Play Performance and Psychosocial Problems	Mean (SD)	Median (IQR)
Play performance total score	157.0 (12.2)	157 (149.0–165.0)
Executive functions	42.7 (5.6)	43 (39.0–47.0)
Environmental context	36.8 (4.0)	37.0 (34.0–39.0)
Play characteristics	41.8 (4.4)	42.0 (39.0–45.0)
Play preferences and interpersonal relationships	35.7 (4.5)	36.0 (33.0–39.0)
Psychosocial problems total score	8.7 (5.5)	8.0 (4.0–12.0)
Emotional symptoms	2.2 (2.2)	2.0 (0.0–3.0)
Conduct problems	1.5 (1.6)	1.0 (0.0–2.0)
Hyperactivity	3.9 (2.7)	4.0 (2.0–5.0)
Peer problems	1.1 (1.3)	1.0 (0.0–1.8)
Prosocial behavior	8.5 (1.7)	9.0 (8.0–10.0)
